# Clinical ethics case consultation in a university department of cardiology and intensive care: a descriptive evaluation of consultation protocols

**DOI:** 10.1186/s12910-021-00668-6

**Published:** 2021-07-23

**Authors:** Andre Nowak, Jan Schildmann, Stephan Nadolny, Nicolas Heirich, Kim P. Linoh, Henning Rosenau, Jochen Dutzmann, Daniel Sedding, Michel Noutsias

**Affiliations:** 1 Institute for History and Ethics of Medicine, Interdisciplinary Center for Health Sciences, Medical Faculty , Martin Luther University Halle-Wittenberg, Magdeburger Str. 8, 06112 Halle (Saale), Germany; 2 Nursing Science Staff Unit, Franziskus-Hospital Harderberg, Niels-Stensen-Klinken, Alte Rothenfelder Str. 23, 49124 Georgsmarienhütte, Germany; 3 Chair of Criminal Law, Criminal Procedure and Medical Law, Law School, Faculty of Law and Economics, Martin Luther University Halle-Wittenberg, Universitätsplatz 6, 06108 Halle (Saale), Germany; 4 Mid-German Heart Center, Department of Internal Medicine III, Division of Cardiology, Angiology and Intensive Medical Care, University Hospital Halle, Martin Luther University Halle-Wittenberg, Ernst-Grube-Str. 40, 06120 Halle (Saale), Germany; 5 Department of Internal Medicine A, Division of Cardiology, Angiology, Nephrology and Intensive Medical Care , Ruppiner Kliniken, Medical School of Brandenburg Theodor Fontane (MHB) , Fehrbelliner Strasse 38 , 16816 Neuruppin , Germany

**Keywords:** Cardiology, Clinical ethics, Ethics consultation, Evaluation research, Intensive care - document analysis

## Abstract

**Background:**

Clinical ethics case consultations (CECCs) provide a structured approach in situations of ethical uncertainty or conflicts. There have been increasing calls in recent years to assess the quality of CECCs by means of empirical research. This study provides detailed data of a descriptive quantitative and qualitative evaluation of a CECC service in a department of cardiology and intensive care at a German university hospital.

**Methods:**

Semi-structured document analysis of CECCs was conducted in the period of November 1, 2018, to May 31, 2020. All documents were analysed by two researchers independently.

**Results:**

Twenty-four CECCs were requested within the study period, of which most (n = 22; 92%) had been initiated by physicians of the department. The patients were an average of 79 years old (R: 43–96), and 14 (58%) patients were female. The median length of stay prior to request was 12.5 days (R: 1–65 days). The most frequent diagnoses (several diagnoses possible) were cardiology-related (n = 29), followed by sepsis (n = 11) and cancer (n = 6). Twenty patients lacked decisional capacity. The main reason for a CECC request was uncertainty about the balancing of potential benefit and harm related to the medically indicated treatment (n = 18). Further reasons included differing views regarding the best individual treatment option between health professionals and patients (n = 3) or between different team members (n = 3). Consensus between participants could be reached in 18 (75%) consultations. The implementation of a disease specific treatment intervention was recommended in five cases. Palliative care and limitation of further disease specific interventions was recommended in 12 cases.

**Conclusions:**

To the best of our knowledge, this is the first in-depth evaluation of a CECC service set up for an academic department of cardiology and intensive medical care. Patient characteristics and the issues deliberated during CECC provide a starting point for the development and testing of more tailored clinical ethics support services and research on CECC outcomes.

## Background

Care of patients with cardiovascular disorders poses regular ethical challenges, especially due to advanced age, cognitive deficits, frailty or multimorbidity [1, 2]. The identification and interpretation of advance stated or presumed preferences regarding potential cardiological treatment are among the more frequent ethical challenges. Furthermore, ethical issues form part of decisions about treatment indication in the form of weighing the potential benefit of a cardiovascular intervention against risks of potential harm. Practical examples of this type of clinical-ethical challenge are the deactivation of the shock function of an implanted defibrillator in a patient with hypoxic brain damage after resuscitation [3, 4] or transcatheter aortic valve implantation in patients with advanced age, cognitive deficits and symptomatic aortic valve stenosis. In the latter case, for example, the risks associated with the intervention have to be evaluated against the benefits, including improvement of the cognitive status after transcatheter aortic valve implantation [5–7].

The relevance of ethical issues in everyday clinical practice is well-documented by several international studies [8–11]. The latter indicate that ethical challenges can have negative effects, such as moral distress for those involved [4, 12–15]. Clinical ethics case consultation (CECC) services have been introduced in many hospitals and other healthcare institutions to provide a systematic and comprehensive analysis of ethical challenges related to patient care in the case of ethical uncertainty or moral conflicts [16–20]. Different models of CECCs exist, however, there are some common features, which are summarised in Box 1.

**Box 1:** Common procedural steps during a CECC [21]

1. Eliciting of medical, nursing, psychosocial and value-related aspects of the case	
2. Definition of the ethical problem(s)	
3. Structured ethical analysis	
4. Identification of possible actions to proceed with in clinical practice	
5. Interdisciplinary decision and recommendation about the next action(s)	

While the possible value of CECC for clinical practice has been stated in numerous publications, there is still little evaluation research related to CECCs [22–24]. Evaluating CECCs by means of empirical methods is important for several reasons. The first is that evaluation may further promote the transparency and accountability of CECCs and those offering this service. A second reason is the potential impact of the results of such studies on the quality of the service. If, for example, service users are dissatisfied with the time which elapses from request to the actual provision of a CECC, this aspect may be reconsidered by CECC providers. A third reason which can be forwarded for the evaluation of outcomes of ethics consultation is that such research may stimulate reflection on the provision of consistent CECC services [23–25].

Little is known so far about the characteristics of CECCs provided within cardiovascular medicine. At the same time, patients with cardiovascular diagnoses frequently give rise to clinical ethics consultation [[Bibr CR26]–28]. This is particularly true for CECCs taking place in intensive care [21, 23]. Information about CECCs in specific clinical contexts is relevant to be able to develop tailored interventions which fit the clinical setting and the needs of patients and health professionals in a particular field of medicine. In addition, such data can inform future evaluation research on the outcomes of CECCs, for example, by pointing out relevant criteria to measure potential benefits or harms of the intervention [21]. Against this background, this paper presents findings of an in-depth descriptive analysis of clinical ethics consultation, conducted by a CECC service, which was set up jointly by clinical ethicists and cardiologists in a German university hospital in November 2018. The aims of this study are

1. To explore the ethical issues underlying requests for CECCs in a cardiology department at a German university hospital.
2. To provide descriptive evaluation data on the content and recommendations of CECCs conducted for patients suffering from cardiovascular diseases, and health professionals.

## Methods

We performed a retrospective document analysis of protocols which were the documented results of CECCs conducted in our department of cardiology and intensive care unit for the period November 2018 to May 2020.

### Setting

The study took place in the cardiology clinic of University Hospital Halle in Germany. The university hospital is a supraregional maximum care provider with 32 clinics/departments with 982 beds. Approximately 40,000 inpatient and 195,000 outpatient treatments are performed per year. The main focus of the University Hospital is on cardiovascular diseases and oncology. The cardiology clinic of the hospital has a ward with 60 beds and an intensive care unit with 25 beds. The medical and nursing team is staffed by: 75 nurses and 60 physicians. The University Hospital has an ethics committee with 35 members from various areas of medical and non-medical care. Membership is honorary. One member is employed on a half-time basis to organise the work of the committee as a manager. A subgroup of the committee (ethics team) located in the Institute for History and Ethics of Medicine conducts CECC services in the cardiology clinic.

### Intervention

The ethics team is composed of two medical ethicists (with backgrounds in philosophy and medical law), a physician (internist and oncologist) and a lawyer (on a demand basis), who have been trained in CECC according to national recommendations [29, 30]. In Germany, CECCs have shown difficulties in getting established or rather getting cases in clinical settings. Therefore, next to ethics consultations performed on request the ethics team and the cardiology department agreed that prompting ethics consultations through a regular weekly reminder was deemed a good measure to remind the health professionals and to reflect on, whether there was a case, which should be discussed as part of CECC.

Thus, the CECC service comprises two modes of initiation:

I. Weekly contact initiated by the clinical ethicist with a senior cardiologist to determine whether there is a possible case for ethics consultation.
II. Ethics consultation on request by members of the cardiology team or by the patients, relatives or surrogates in case of need.

The weekly request (I) is characterised by a standardized e-mail request sent by the committee manager to the same senior cardiologist at the same time each week asking if there is a need for consultation. The senior cardiologist explores whether there is a need for consultation in the regular team meetings with the other senior physicians, who forward the question to the ward team (top-down principle). The requests are bundled and sent to the senior cardiologist, who then forwards them to the ethics team.

Spontaneous inquiry (II) is possible at any time by any staff member of the clinic, and by patients and their relatives. Information and contact details are available on every ward of the hospital (bottom-up principle). Requests can be made informally at any time.

The ethics team can be contacted via telephone, an emergency cell phone, e-mail or the patient management system. At least one and a maximum of three consultant(s) from the ethics team will participate in each consultation. The selection of team members for each case is based on time capacity and experience with the subject matter. If difficult legal issues are identified in advance, the participation of a lawyer is offered. The committee manager participates in each consultation. Subsequent to both ways of initiating a CECC, a first meeting between a consultant, physicians and nurses of the respective ward will be arranged by the committee manager usually within 24 h of the request. This session takes place on the patient’s ward and follows a structured process, which is illustrated in Box 1. The initial session usually takes about 20–45 min. In addition, the ethics consultant visits the patient and eventually proxies or relatives to elicit and discuss her or his preferences and values regarding the questions at stake if possible. Furthermore, cases are always discussed between at least two members of the ethics team. Half-yearly debriefings of selected cases are held with the ethics committee for quality assurance purposes.

**Fig. 1:**
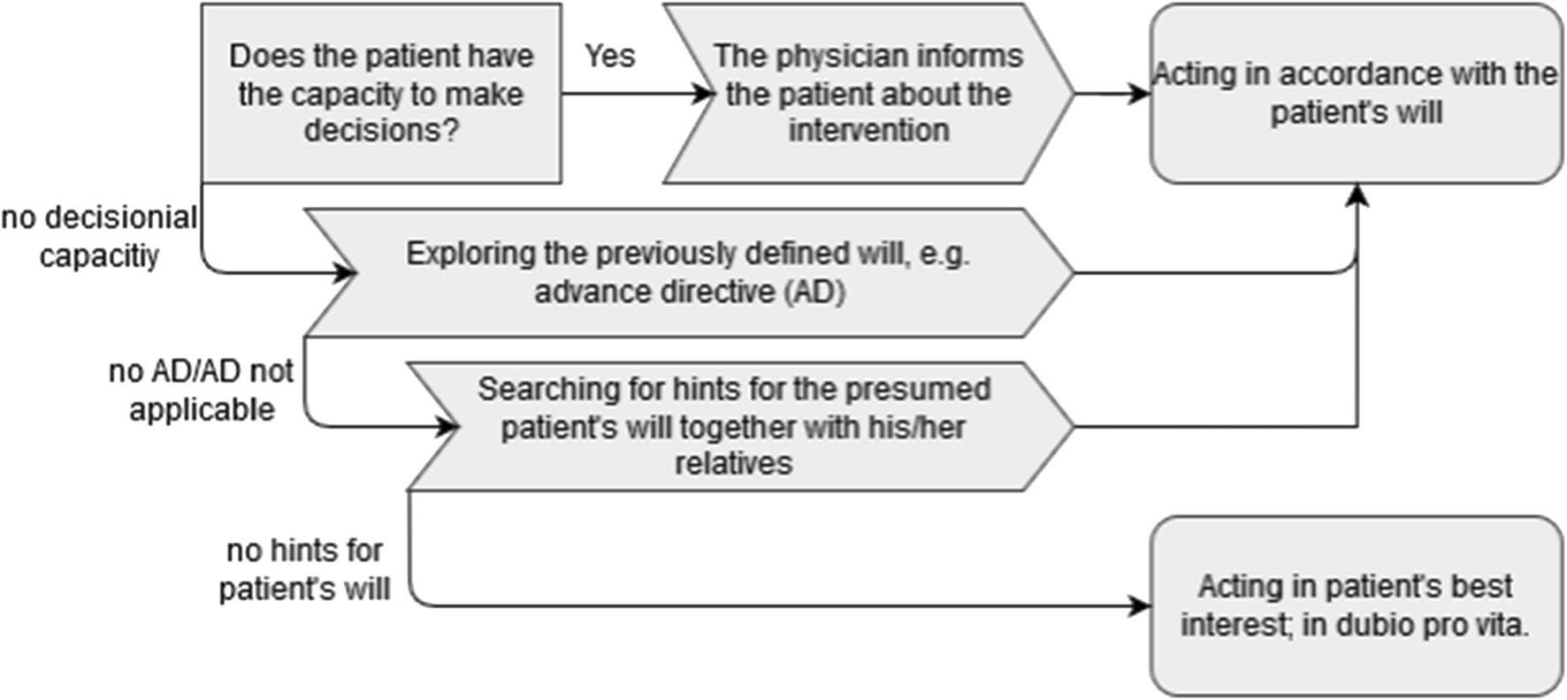
Schematic summary of the procedural steps regarding evaluating the patient’s will as part of the CECC

The respective indication (1) and the patient’s will (2) as a representation of autonomy are the focus of the CECC. Regarding the indication, the main task of the CECC is to identify the rationale for offering (or limiting) diagnostic and/or therapeutic procedures with a focus on the value judgments underlying the formulated goal of care and the weighing of benefits and harms. If a treatment is not or no longer indicated from a medical point of view, it is not permissible under German law and must be discontinued. However, a large number of publications in Germany show different definitions of the term medical indication, leading to ethical uncertainties in practice [31–33]. Only treatments that are actually indicated may be offered to the patient for consent. Therefore, the patient’s will regarding possible treatments is limited to those treatments indicated. Informed consent is legally and ethically required in Germany for all medical treatments. The legal requirements for the patient to be able to give informed consent are that he or she has the capacity to give consent (decisional capacity). This means that the patient is able to understand the significance and scope of the medical intervention, evaluate the pros and cons, and communicate the decision. It is the task of the attending physician to determine this [34]. If the patient is not competent, a proxy must give informed consent for him/her based on the patient’s will [[Bibr CR35]]. Regarding the evaluation of the patient’s will, the procedural steps are summarised in Fig. 1.

If necessary, the ethics consultant, in collaboration with further stakeholders of the process, initiates further steps, such as additional discussions with the patient’s representatives, the nursing home staff, or the involvement of relevant specialists (e.g. cardiac surgery, geriatrics, palliative care). In addition, legal advice from experts in medical law is sought if required. Subsequent to the ethics consultation process, a draft summary including the recommendations of the clinical ethics team is sent to the healthcare team for comments and it is documented in the patients’ record after completion. This is, firstly, to make the conversation and the recommendations transparent and, secondly, to prevent misunderstandings related to identified, ethically relevant but also clinical issues. The attending physician informs the patient (if possible) and his or her relatives or legal representatives, if they were not present at the consultation, of the contents and recommendations of the CECC. The possibility of a renewed discussion is always offered. Access to the medical record and the CECC protocol by the patient or the patient’s proxy is always guaranteed.

### Data material and analysis

A semi-standardized protocol usually comprising two to five pages, was used for the documentation of each case. It consists of (a) the patient’s name, (b) a list of important relatives, (c) a list of members of the treatment team, (d) the ethical question(s), (e) diagnoses, (f) medical-nursing aspects, (g) the patient’s will/decisional capacity, (h) the procedure(s) discussed or medically indicated and (i) the CECC recommendations. Furthermore, we had data on the duration of the consultation, time from request to recommendation, and time from recommendation to discharge. Data was anonymized regarding the names of the CECC participants. All protocols from November 2018 to May 2020 were analysed.

We descriptively analysed diagnoses, the status (i.e. professional, patient, relative) of the requester of the CECC, the profession of the CECC participants, the clinical (ward, decisional capacity, length of stay) and patient’s socio-demographic characteristics (age, gender), the duration of consultation, the time from request to recommendation and the time from recommendation to discharge. We report the data with absolute and relative frequencies and the median and minimum to maximum ranges because the data were not normally distributed.

To be able to elicit also more implicit aspects of CECC we employed a structured qualitative content analysis approach based on hermeneutics [36]. Two researchers, one with a background in medical ethics (AN) and the other in philosophy (NH), inductively coded the first eight protocols independently and derived a category system for the ethics questions, decisional capacity, the patient’s will and recommendations. In addition to small wording differences, the researchers had formed identical categories because the densely written protocols left little room for interpretation. The categories were, therefore, briefly discussed in the research team and, subsequently, the rest of the cases were coded. No further codes were necessary in the process. MAXQDA 2020 was used for the analysis. Due to the lack of interpretative depth, the categories were reported quantitatively with absolute and relative frequencies. See Table 1 for information on the coding for the quantitative reporting. Additionally, we use summarised case descriptions as examples to illustrate the CECC process.

**Table 1:** Codes created for the quantitative evaluation

Protocol category	SPSS variable	Exemplary values
List of important relatives	Status of relatives_1 (ascending number for each relative)	Sister, brother, husband, wife, …
	Legal guardianship_1	Yes/no
	Participated in CECC process_1	Yes/no
List of members of the treatment team	Members requesting CECC_1 ascending number for each member)	Nurse, physician, …
	Participated in CECC process_1	Nurse, physician, …
Ethical question	Reasons for request_1 (ascending number for reason)	Balancing benefit and harm, eliciting patient’s will
Leading diagnoses	Diagnoses_1 (ascending number for each diagnosis)	Coronary heart disease, sepsis, …
Patient’s will	Advance directive	Available/not available
	Applicability of advance directive	Yes/no
Decisional capacity	Decisional capacity	Always present, not present, sometimes present
Medical-nursing aspects	Ward	Ward X, Y, Z, …
	Gender	Female, male, diverse
	Age in years	X years
	Length of stay in the hospital	X Days
Recommendations	Recommendations_1 (ascending number for each recommendation)	Change to palliation, implementation of treatment, continuation of treatment,…

## Results

Thirty-seven consultations took place throughout the entire university hospital during the study period. Of the 37 consultations, 24 (64.86%) were requested by the cardiology clinic and were, thus, included in the document analysis. Eighteen inquiries resulted from the regular weekly contact between the ethics consultant and the senior physician of the department. A CECC was requested by one of the wards of the department in a further six cases. The CECCs were initiated by physicians of the department in 22 cases, once by a general practitioner of the patient and once by a relative of the patient. The time between request and initial meeting between the ethics consultant and a member of the healthcare team was less than 24 h, with two exceptions. The median time of the CECC from the initial meeting to the recommendation was 1 day (R: 0–7 days). Two patients died prior to the initial meeting and one patient discharged himself against medical advice prior to the consultation.

Physicians were involved in all cases during the CECC process, which encompassed 1 to 4 meetings with different stakeholders including the patient. Nurses of the ward participated in 18 cases and physicians of other disciplines (geriatrics [n = 2], palliative care [n = 2], oncology [n = 1]) participated in 5 cases. The patient’s relative(s) were involved in 13 cases, of which 8 were legal representatives of the patient. In 6 cases, the patient was represented by a professional legal guardian. In 2 cases, the staff of the nursing home where the patient lived were contacted to obtain relevant information.

### Medical, socio-demographic data and procedural aspects

At the time of the CECC, 13 patients (54.2%) had been treated on a general ward, and 11 patients (45.8%) on an intensive or intermediate care unit, respectively. Fourteen patients (58%) were female, and the median age was 79 years (R: 43–96). Twenty patients (83%) were lacking decisional capacity continuously, and the decisional capacity in one case was fluctuating. In seven of these 21 cases, verbal communication was possible but these patients were deemed not to be able to make a decision according the requirements of decision capacity. The median length of stay prior to request was 12.5 days (R: 1–65 days). Table 2 provides an overview of the length of stay and CECC-related milestones.

**Table 2:** Length of stay and CECC-related milestones

Patient’s length of stay in the hospital in median days (min–max) (n = 24)	Time from patient’s hospital admission to request of the CECC in median days (min–max) (n = 24)	Time from CECC request to CECC performance in median days (min–max) (n = 21)	Time from the request of the CECC until recommendation in median days (min–max) (n = 21)	Time from the CECC to patient’s discharge in median days (min–max) (n = 21)
19.5 (1–65)	12.5 (1–65)	0.5 (0–3)	1 (0–7)	4 (0–18)

min–max: Minimum to maximum days

The diagnosis leading to hospital admission was a cardiovascular disease in 14 cases (58%). Cardiology-related diagnoses were the most frequent main diagnoses (n = 29), followed by sepsis (n = 11) and oncological diseases (n = 6) (multiple diagnoses possible). The majority of patients were characterised by multimorbidity. As a result, even supposedly trivial diagnoses in combination with the other diagnoses could lead to complex situations regarding clinical decision-making.

### Reasons for the request and content of CECC

#### Reasons for request

Uncertainty about the balancing potential of benefit and harm related to the medically indicated treatment was the main reason for a CECC request in n = 18 (75%) cases (multiple reasons possible). Factors contributing to the uncertainty were advanced age, limited life expectancy, multimorbidity and frailty (according to the clinical judgment). Incongruent perspectives of the relatives and ward staff regarding the choice of treatment were the reason for the initiation of a CECC request in many cases (n = 16). Furthermore, differing views between health professionals and patients (n = 3) or between different team members (n = 3) concerning the best individual treatment option led to a CECC. One further reason was the uncertainty of the treatment team, relatives and/or proxies regarding the patient’s supposed will regarding a particular intervention in the case of lacking capacity (n = 6). Additional challenges leading to CECC requests were situations in which no close relatives of the patient could be located or the statements of the respective patient and/or the relatives were incongruent.

The following case summary provides an example of a typical scenario leading to a request for CECC.

Example Protocol Y7:

An 84-year-old female patient was resuscitated outside the hospital. From this time on, she had to be ventilated. A tracheotomy and a percutaneous endoscopic gastrostomy (PEG) tube are medically indicated to ensure long-term ventilation and nutrition. The patient’s two sons and one daughter-in-law make contradictory statements regarding the mother’s presumed will. A legal guardian has been appointed for the patient. He does not know the patient and does not feel able to make this decision. A CECC was requested to support the elicitation of the patient’s (presumed) will in light of the treatment options offered and related goals of care. In order to find out more about the patient’s will, the CECC suggested contacting the patient’s nursing home and the nurse who had been caring for the patient for years.

#### Content of the CECC

**Fig. 2:**
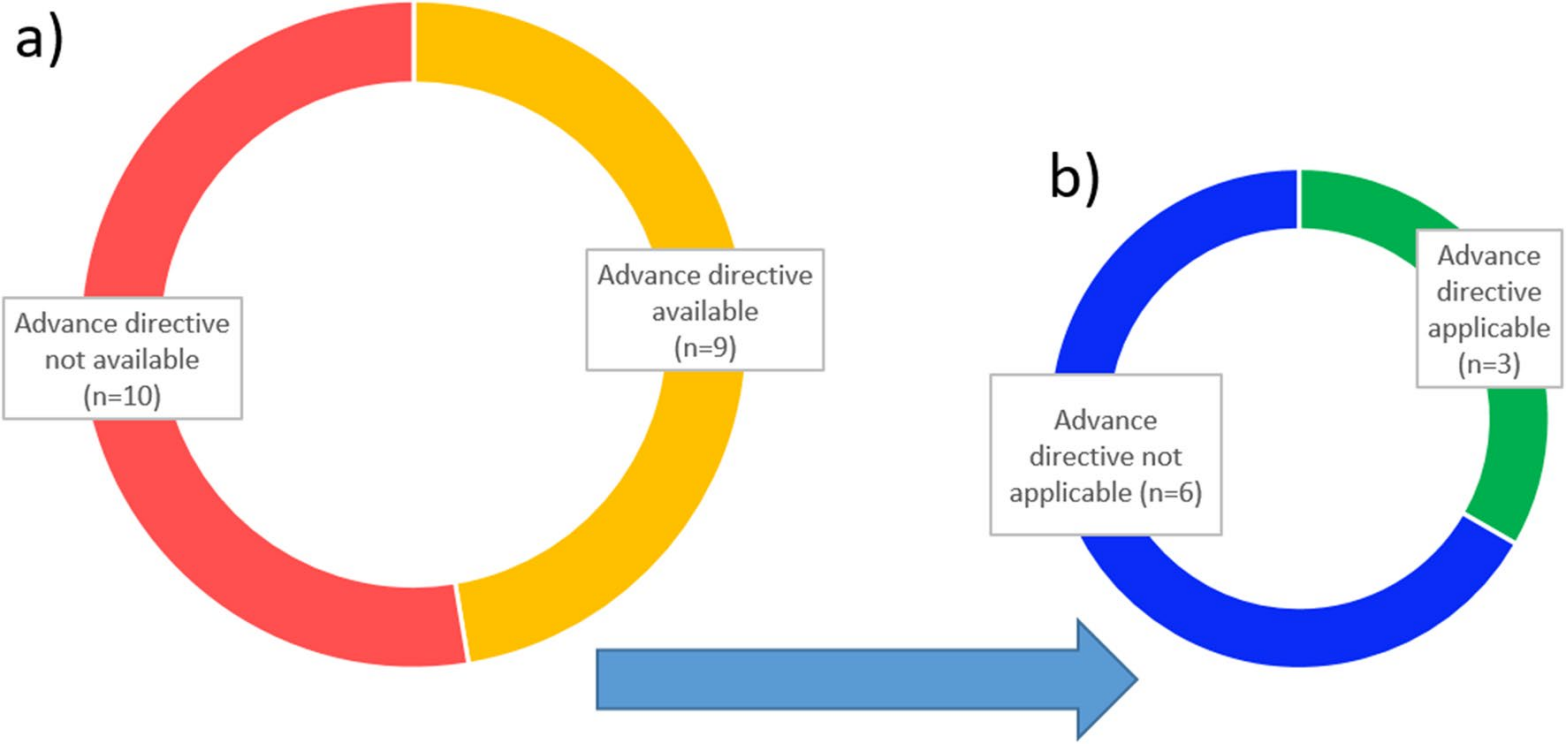
Summary of the role of advance directives in the CECC. **a** Availability of an advance directive for patients incapable of consenting during the CECC (excluding two patients deceased before consultation). **b** Evaluation of the applicability of the existing advance directives to the specific situation regarding CECC participants

In accordance with the method utilised in the CECC, the analysis of protocols showed that the centre of discussion during the CECC was most often the *patient’s* *will*, on the one hand, and the rationale for *medical indication*, in particular the benefits and harms of treatment as evaluated by the healthcare team, on the other hand. Advance directives were available regarding the first pillar of ethical decision-making in nine cases. In the latter, life-sustaining treatments, such as artificial respiration and resuscitation, were refused. However, these orders were always linked to specific situations or presumed treatment outcomes (e.g. permanent severe need for care or irreversible brain damage). In three of these cases, advance directives were directly applicable to the situation (see Fig. 2). In six of the nine advance directives, the situation or outcome described could not be identified with certainty by the treatment team. In these cases, the patient’s will had to be clarified via other sources (see Fig. 1). The non-applicable advance directives were included in the considerations when determining the patient’s (presumed) will. In all cases in which a patient was deemed to have no decisional capacity, treatment options were discussed with the patient as far as feasible.

The CECC focused mostly on the balancing of (presumed) benefit and harm in discussions about the *medical indication*, as a prerequisite for any offering of a diagnostic or therapeutic procedure. Based on an explicit discussion of value judgments in the context of the medical indication, the treatment options considered were withdrawn in three cases because there was no longer an indication due to various characteristics of the patients. This may be the case if, for example, prolongation of life by treatment no longer seems possible from a medical point of view due to the multimorbidity overall condition of the patient. Alternatively, the risk of dying earlier as a result of the treatment is much higher than the possibility of prolonging life by the treatment. The following case summary provides an illustrative example for such a scenario.

Example Protocol N2:

A patient with dehydration, severe hypernatremia, advanced dementia and an autoimmune skin disease that has also spread into the mouth refuses to eat and take oral medication. The treatment team considers the placement of a PEG tube is indicated. In the course of the CECC, a geriatrician was consulted who pointed out that the refusal of oral intake was compatible with the advanced state of dementia. Furthermore, it was stated that the application of a PEG tube at this stage of dementia, also based on existing recommendations in guidelines, was deemed to be contraindicated due to a lack of benefit and the associated potential harm of the procedure for the patient at this advanced stage of the disease. As a result of the CECC, the placement of PEG was not further deemed as indicated in the respective patient.

### Recommendations as an outcome of the CECC process

According to the CECC protocols, consensus between participants could be reached in 18 out of 24 consultations (75%). The dissent could not be resolved in three cases. Two patients died before a final recommendation could be reached and one patient discharged himself prior to completing the CECC process. Recommendation usually included practical steps relevant to further care and treatment. The implementation of a disease-specific treatment intervention was recommended in five cases. A confirmation of palliative care and limitation of further disease-specific interventions was the therapeutic goal recommended by the CECC in 12 cases. The CECC team decided on a ‘freezing’ of the current treatment with a re-evaluation in the near future in four cases (see Table 3).

**Table 3:** Recommendations of the CECC

Recommendation at the end of the consultation	n = 21
Change of treatment goal to palliation (and limit of disease-specific interventions)	12
Implementation of disease-specific treatment	5
Confirmation and continuation of the medical treatment indicated with a predefined plan for re-evaluation	4

Recommendations were based mostly on the (presumed) will of the patient. The absence of evidence of the patient’s will in two cases led to the recommendation to perform life-saving treatment (*in dubio pro vita*). Recommendations were based on the lack of medical indication in four cases, following a discussion during the CECC with participating health professionals regarding the benefits and harms of possible technical interventions.

Example Protocol T6:

A 54-year-old patient with hypoxic brain damage after about 45 min of cardiopulmonary resuscitation with still unclear neurological prognosis. The treatment team is uncertain whether continuation of intensive care treatment is in accordance with the patient’s will. The patient relies on mechanical ventilation. A legal representative could only locate the patient’s sister, who had not had contact with the patient for years and could not provide reliable information about the patient’s will. Due to the lack of information about the patient’s will, the continuation of medically indicated life-sustaining treatment was recommended by the CECC.

The analysis of the protocols showed no differences regarding the points presented between the consultations initiated by the weekly prompts by the ethics team and those from requests from the members of the cardiology team.

## Discussion

This paper provides an in-depth descriptive analysis of CECCs in cardiology and intensive care in internal medicine. To the best of our knowledge, this is the first publication with qualitative and quantitative data elicited from CECC protocols from an academic department of cardiology and intensive care. In the following we will discuss the findings with a focus on the common reasons for CECC requests in cardiology, procedural aspects of the CECC service provided, and implications for the development of more tailored clinical ethics support interventions and respective evaluation research.

### Requests for CECCs in cardiology and intensive care

The requests for CECCs reflect the technical possibilities provided by modern cardiology and intensive care and the associated uncertainty about the benefits and harms of the medical interventions available in a subgroup of patients characterised by advanced age, limited life expectancy, multimorbidity and frailty. Given these challenges associated with decisions about the offering of specific interventions, it is not surprising that the majority of CECC requests were made by physicians involved in the treatment of patients. By contrast, only few requests had been made by nurses or patients’ representatives or relatives. The reasons for this may be the focus of the CECCs on ethical issues related to decisions about the medical treatment and less about ethical challenges related to nursing care. In addition, there may be a low public awareness of CECCs or a lack of information about CECCs in the department [8, 37–39]. Furthermore, due to the specific approach of contacting a senior cardiologist on a weekly basis, most CECCs occurred as a result of prompting the physicians with a question about a possible need for a CECC. The fact that most consultations stem from regularly prompts on behalf of the CECC team may point to the potential of such initiatives to increase the awareness of ethics issues [40–42] and, subsequently, increase the number of CECCs [43]. Given data on the experience of nurses with ethical challenges [44–46], one complementing strategy could be a joint weekly call with a senior nurse to include this professional perspective as well.

### Clinical-ethical issues in CECCs provided to cardiology and intensive care

Our analysis reveals that, comparable to other studies, CECCs in cardiology and intensive care focused on eliciting the patient’s will and the balanced multidisciplinary discussion of potential benefits and harms [23, 24, 47]. Our findings suggest that there was often no dispute about facts but about the judgement of pivotal issues related to the individual case (i.e. considering advanced age, multimorbidity, frailty, residual quality of life and limited life expectancy). If this value judgement is incorporated implicitly into the supposedly objective medical view, there is a risk that a patient may be deprived of decisions which take into account personal values. In this respect, our analysis shows that one important function of a CECC was to make explicit value judgments regarding (presumed) benefits and harms [48]. It is important here to distinguish harm and benefit viewed by health professionals and evaluations in this respect on side of the patient. In line with a principle based approach [49] we would argue that health professionals do have a task to evaluate benefit and harm but must be clear about their evaluation as well as the reference point for the judgement. One such reference point are considerations according to the “best interest” [50] of the patient. This is also an important legal point. If a therapy is discontinued due to a lack of indication, this may only happen based on medical considerations but not on the basis of considerations on the part of the treatment team regarding their values. If the medical indication is given, it is the patient’s values and wishes, which are decisive about whether the treatment is taking place or not. During the process of deliberation offered by a CECC, participants were able to exchange their perceptions and views and try to find a common ground. In parts of the cases, involvement of disciplines outside cardiology contributed to an informed weighing of benefits and harms. In this case, the CECC provided a forum for deliberation about all options medically available, ranging from disease-specific advanced cardiovascular interventions to palliative care. As evidenced by our analysis, in the case of uncertainty about a patient’s will and the balancing of benefits and harms, an ‘*in dubio pro vita*’ approach was chosen. Furthermore, the change to palliative care goals as a result of CECC discussions reflects that cardiology and intensive care might benefit from models of early palliative care, which have already been established in some leading centres for patients with heart failure [51–53].

### Time and procedural characteristics of the CECC service in cardiology and intensive care

Our data show that clinical-ethical uncertainties were usually resolved and a consensus reached within an average of 48 h. In contrast to the prejudice that the CECC is too time-consuming [8, 54], our results instead point to the fact that the CECC can facilitate a swift process in otherwise time-consuming decision-making processes characterised by delays due to dissent and uncertainty [22]. In light of the known substantial increase of the workload in cardiology and intensive care [55], handling of communications and appointments with the patients’ relatives or legal guardians and the documentation process of the CECC undertaken by ethics professionals might aid in relieving the cardiovascular and intensive care specialists from these time-consuming activities. However, a critical reflection on the CECC in an environment with a high case load and time constraints in cardiology is still important. Time is a crucial but also ambivalent factor in the joint decision-making process of CECCs. Waiting for the development of a health status during an already initiated intensive treatment, for example, can provide a better foundation for the prognosis but, at the same time, can place an increased burden on the treatment team, the relatives and, last but not least, the patient [56].

A procedural characteristic of the CECC provided is the close collaboration of the ethics consultants with experts in medical law. Such a collaboration provides several advantages: firstly, it creates legal certainty for the consultation by eliminating unlawful options and clarifying the legal framework within which to operate. Secondly, it adds another perspective to the determination of the patient’s (presumed) will, not only by laying out the legal guidelines for this determination and the requirements for advanced directives but also by providing help for the interpretation of the patient’s statements. While the final decision and the (legal) responsibility remains with the treating physician, joint ethico-legal consultation can help to prevent legal conflicts that eventually might also have an impact on the treatment of the patient.

### Possible implications for a tailored CECC and evaluation

The preceding analysis points to more general but also more specific features of CECCs in a university cardiology department, which may serve as a starting point for the development of more tailored CECC interventions and suitable outcome criteria. The comparison of the number of CECCs from the cardiology clinic, the only clinic where the proactive weekly request was implemented, with the rest of the university hospital (without a proactive approach) suggests that weekly inquiries may also lead to an increased use of the ethics offer. This could be due to the fact that this regularly draws attention to the offer. Another point could also be that the involvement of leading personnel (such as a senior physician in our study) could lead to a higher acceptance of CECCs by the rest of the team. On the part of the consulting team, special attention must be focused on avoiding an authority bias. Regarding the intervention, the length of time prior to a request for a CECC raises the question whether more proactive interventions, for example, within the context of usual ward rounds, may facilitate an earlier analysis of ethical and sometimes legal issues. This is particularly the case given the high proportion of patients lacking decisional capacity in our sample. Treatment of these patients regularly begs questions regarding presumed or advance stated will and weighing benefits and harms from a professional perspective. Advance directives can help to record the patient’s will for future situations and the need for consideration by relatives or the treatment team. Unfortunately, our research shows — similar to other studies (for a review see Fagerlin and Schneider [57]) — that advance directives often fail to have an effect in practice. One reason for this is that they are often not applicable to the specific situation. This can frequently be the reason for ethical problems. The implementation of an advance care planning offer could counteract these challenges in the future as a kind of a preventive ethics offer in hospitals. One further aspect of future more tailored interventions are shorter and more focused elements of ethics support instead of the provision of a comprehensive CECC which often takes up to one hour of time involving several health professionals. A time investment which needs to be justified in light of competing tasks which benefit patients. Reflections of modifications regarding CECC services should be also aligned with considerations regarding adequate evaluation criteria. In this respect, a detailed descriptive account of procedurals steps of different modes of ethics support, related goals and expertise which are contributed by means of a particular CECC service is a prerequisite [21]. Furthermore and in light of the findings of this descriptive study, the more specific features of CECCs in cardiology should be taken into account when defining appropriate evaluation criteria. Possible candidates for such context-specific features are the range of technical measures and interventions offered as part of cardiology care and the challenges associated with prognosis and different end-of-life trajectories in this field of medicine.

### Limitations

The CECCs reported were performed with a limited number of cases only at a single centre, therefore, results are per se not generalizable. However, our results regarding the ethical issues and requests are similar to those of other studies [28, 58, 59] and add to our knowledge on CECCs in the particular setting of cardiology and intensive care. We based our analysis only on retrospective protocol information and, thus, we cannot a) determine the overall of quality of the CECCs performed since document analysis alone is hardly capable of reflecting all levels of quality, and b) infer firm causal conclusions between CECCs and possible outcomes. Furthermore, our analysis may have been influenced by data interpretation bias since two of the authors are members of the CECC team. We tried to increase trustworthiness of the analysis through the reporting of ‘negative’ findings, for example, dissent at the end of a CECC, illustration through case summaries and discussion of the results in the research team. The protocols analysed were outcome protocols and, therefore, already written very densely and briefly, leaving little room for interpretation in terms of coding.

## Conclusions

The combination of a range of technical interventions and a subgroup of patients with advanced age, multimorbidity and frailty is one trigger for ethical uncertainty and conflicts in cardiology and intensive care. A CECC can be a viable intervention in facilitating discussion on these issues between different stakeholders with and on behalf of the patient. The timely initiation of ethics consultation through direct contact with a senior cardiologist proved to be a good strategy to raise ethical issues in daily cardiology practice and could be extended to contact with nursing staff. The evaluation of this intervention based on document analysis can give a first insight into the topics, processes and quality of CECCs. Further analysis with mixed-methods approaches and different sources is needed to gain a clearer picture of the quality domains of the structure, process and outcome and the tailoring of CECC interventions in cardiology.

## Data Availability

The datasets generated and/or analysed during the current study are not publicly available due to confidentiality reasons but are available from the corresponding author on request.
